# Combating Gaming Disorder in 2024: A Survival Manual

**DOI:** 10.1192/j.eurpsy.2024.823

**Published:** 2024-08-27

**Authors:** D. M. Ribeiro, D. Teixeira

**Affiliations:** Departamento de Psiquiatria e Saúde Mental, Centro Hospitalar Universitário do Algarve - Hospital de Faro, Faro, Portugal

## Abstract

**Introduction:**

Gaming Disorder (GD) has not been officially recognized as a diagnostic entity in the DSM-5, being listed in the “conditions for further study” section. However, it is described in the ICD-11, and clinically, it is observed that an increasing number of individuals, particularly the younger population with easier access to technology, are affected by this issue. Nefarious consequences include loss of performance at school/work and a potential for failing other responsibilities such as in the familiar and social spheres.

**Objectives:**

Despite its harm, psychiatrists are generally less familiar with this entity when compared to other psychiatric disorders. Thus, our main goal was to establish a comprehensive and hollistic review of its approach.

**Methods:**

A bibliographical research on the topic was conducted from the available scientific literature on the topic, with the utmost priorization of evidence-based sources.

**Results:**

The overall prevalence of Gaming Disorder is challenging to assess precisely, but it is estimated to be around 3%, making it comparable to obsessive-compulsive disorder and some substance use disorders. It is more common than pathological gambling. Clinically, GD is characterized by an excessive preoccupation with gaming that supersedes all aspects of life. It may also involve a compulsion to play and the presence of withdrawal symptoms from periods when there are no gaming activities. The behavior is driven by the ACE triad (anonymity, convenience, and escape). Often, individuals with GD do not seek treatment. Although there are no specific pharmacological agents, antidepressants, mood stabilizers, and naltrexone have shown some success. In psychotherapies, cognitive-behavioral therapy has the strongest evidence.

**Conclusions:**

There is a limited amount of information on GD, and when researching the topic, one primarily encounters information on other substance-related addictive disorders and, in the case of behavioral disorders, gambling. However, as young people are increasingly exposed to screens and video games with potential harmful effects on their development, and in adults, inhibiting them from taking on work and family responsibilities, it is essential to conduct more studies on the subject to prevent these deleterious consequences.

**Disclosure of Interest:**

None Declared

